# Can we predict which patients with plantar heel pain are more likely to benefit from insoles? A secondary exploratory analysis of a randomized controlled trial

**DOI:** 10.1186/s13047-022-00516-9

**Published:** 2022-02-10

**Authors:** N. Rasenberg, L. J. M. Dijkgraaf, P. J. Bindels, S. M. Bierma-Zeinstra, M. van Middelkoop

**Affiliations:** Department of General Practice, Erasmus MC, University Medical Center, PO Box 2040, 3000 CA Rotterdam, The Netherlands

**Keywords:** Plantar heel pain, Insoles, Prognosis, Patient characteristics

## Abstract

**Background:**

Plantar heel pain (PHP) is a common cause of foot complaints, for which treatment with custom-made insoles is frequently applied. So far few studies have investigated patient characteristics that predict response to these treatments. The aim of this secondary exploratory analysis was twofold; firstly, to identify patient characteristics that predict prognosis in patients with PHP treated with insoles, and secondly to identify characteristics that might interact with treatment with insoles.

**Methods:**

Data from a randomized trial in which participants received either custom insoles (*N* = 70) or sham insoles (*N* = 69) were used. At baseline, information was collected on demographics, foot symptoms, foot and ankle range of motion, navicular drop, presence of neuropathic pain, physical activity and other illnesses in the last 12 months. The primary outcome of this study was the Foot Function Index score (FFI) at 26 weeks. Multivariable linear regression models were generated to identify patients characteristics that predict the outcome for each type of intervention (i.e. insoles and GP-led usual care).

**Results:**

We found two variables associated with a better function score at 26 weeks in patients treated with insoles, female sex (β − 9.59 95%CI -17.87; − 1.31) and a lower FFI score at baseline (β 0.56 95%CI 0.30; 0.82). Explorative analyses in patients treated with insoles showed no significant interaction effects between the type of insole (custom-made versus sham) and any of the potential predictive factors.

**Conclusion:**

When communicating about the effect of insoles for PHP clinicians should take sex and the amount of pain and disability at first presentation into account. Women and people with better foot function scores at baseline (according to FFI) might respond better to treatment with insoles in terms of foot function.

**Trial registration:**

Trial registration: NTR5346.

## Background

Plantar heel pain (PHP), is most commonly defined as pain located at the anteromedial part of the plantar heel during weight-bearing when plantar fascia pathology has not been confirmed by imaging [1, 2]. The incidence in the Dutch general practice is estimated to be 3.83 per 1.000 person-years, where the average general practitioner (GP) sees approximately 8 new cases each year [3]. Several risk factors for developing PHP have been identified in literature, including a high BMI, limited ankle dorsiflexion, being a runner, work-related weight-bearing activities and being female [4–8]. The long term prognosis of PHP is unknown, as the duration of complaints of PHP varies greatly among studies [1, 8, 9]. PHPhas a significant negative impact on quality of life (both in terms of general and foot specific health) [10]. Most reported complaints are difficulties carrying out work-related activities and sports activities [11]. Treatment strategies vary greatly among GPs and multiple interventions are often applied in patients with PHP during the course of their complaints [3]. Of the many treatments options available, orthotic devices, such as insoles, are one of the most commonly prescribed interventions [3, 12]. Though, evidence on the effectiveness of insoles in patients with PHP is conflicting [13–15]. With Whittaker et al. finding: “moderate-quality evidence that foot orthoses are effective at reducing pain in the medium term” [14]. And Rasenberg et al. concluding: “Foot orthoses are not superior for improving pain and function compared with sham or other conservative treatment in patients with PHP.” [16] A recent best practice guideline based on systematic review of literature and clinical expert reasoning found: “good agreement to ‘step care’ using custom foot orthoses for general pain in the short term.” [17].

Little is known about factors that might predict recovery or response to treatment with insoles in patients with PHP. Whittaker et al. recently found: “People with plantar heel pain who use foot orthoses experience reduced foot pain if they have greater ankle dorsiflexion and lower BMI, while they experience improved foot function if they have lower fear-avoidance beliefs and lower BMI.” [18] Whereas, Wu et al. identified several physical measures that if present increased the rate of positive outcome (in terms of pain function or recovery) in patients that received custom foot orthosis for PHP [19]. Though, more studies focusing on predicting response to treatment using data from high-quality studies are needed. A recent randomized controlled trial from our group found that GP-led usual care seemed to be slightly more beneficial than treatment with custom-made insoles and found no differences between custom-made insoles and sham insoles [15]. However, some patients did report an improvement after receiving insoles, while others did not. In this trial no differences were found between custom-made insoles and sham insoles and blinding between these two groups was successful [15]. This means that the context for patients in these two groups was identical and that the data from these patients is suitable to assess the prognosis of PHP in patients treated with insoles as an overall group. Therefore, the aim of this secondary exploratory analysis was twofold; firstly, to identify patients characteristics that predict the long-term complaints of patients with PHP treated with insoles, and secondly to identify characteristics that might interact with insole treatment response.

## Methods

### Population

For this secondary analysis, data from a previously published randomized trial investigating the effectiveness of treatment with custom-made insoles in patients with PHP were used [15]. PHP was characterised as pain at the medial hind foot, reproducible on palpation. Inclusion criteria for PHP were: age between 18 and 65 years, minimal pain duration of 2 weeks and presentation with PHP, characterised as pain at the medial hind foot, to a GP or sports physician. Exclusion criteria were recurrent complaints of PHP for more than 2 years, complaints caused by trauma, earlier treatment for PHP by a podiatrist or with insoles, suspected (by the GP or sports physician) (osteo) arthritis in the subtalar or talonavicular joint, suspected tarsal tunnel syndrome, suspected stress fractures, infections or tumours in the painful foot, presence of systemic diseases (such as ankylosing spondylitis, psoriasis or multiple sclerosis) and insufficient understanding of the Dutch language. The trial adhered to the principles of the Declaration of Helsinki and the medical ethical committee of the Erasmus Medical Centre has approved the study (MEC: 2015–253) and was prospectively registered at the Dutch Trial Registration (NTR5346) [20]. Patients between ages 18–65 years, that presented themselves at their GP with PHP and with a minimum of 2 weeks of complaints were eligible for this trial. A total of 185 patients were included, after informed consent was received. For the current study, only participants who received an insole treatment were included.

### Interventions

Patients allocated to the custom-made insole group were referred to a podiatrist and randomly allocated to receive either a custom-made insole or a sham insole. The custom-made insole was produced according to the standard practice of their podiatrist (*n* = 70). The purpose of the custom-made insole was to influence the biomechanical process to reduce traction on the plantar aponeurosis and to reduce ground reaction force below the tuber calcaneus. The sham insoles were produced according to a standardized procedure, all by the same podiatrist (*n* = 69). The sham insoles were designed to provide as little mechanical effect as possible, while having a visual effect similar to that of a custom insole. The patients were blinded to the type of insole they received. All patients received an information booklet containing general information on PHP including stretching and strengthening exercises targeted at PHP [4, 16]. Further details of the STAP study, and on the procedures mentioned here, are described elsewhere [13, 15].

### Measurements

At baseline all participants filled in a questionnaire containing questions regarding demographics: age, sex, weight and length (to calculate body mass index (BMI)); general health status: musculoskeletal pain other than in the foot, self-reported illnesses in the last 12 months; foot complaints: affected side (right, left or bilateral), duration of complaints, pain severity (11-point numerical rating scale (NRS), including first step pain, pain in rest and during activity), neuropathic aspect of the pain according to the Doleur Neuropathique 4 (DN4 (range, 0–10)) [21], the Foot Function Index (FFI (range, 0–100), higher score indicates more disability/worse function) [22] and questions regarding lifestyle factors: physical activity (Short QUestionnaire to ASsess Health enhancing physical activity (SQUASH) questionnaire) [23] and standing work for a prolonged time (4-point Likert scale). For further analysis, participants, who indicated ‘bilateral’ as the affected side (*N* = 32), were randomly assigned to either ‘left’ or ‘right’.

Patients that were referred to a podiatrist underwent a standardized physical examination, including the range of motion in the tarsometarsal joint and the first metatarsophalangeal joint (MTP-I) as measured with a goniometer (number of degrees), the navicular drop test (millimeters) [24] and the posture of the foot using the standardized foot posture index (FPI) of both feet [25]. The podiatrist was blinded for participant allocation while performing these measurements.

At 12 and at 26 weeks participants received questionnaires containing questions on foot function (FFI), self-reported recovery (7-point Likert scale) and pain severity in rest, during activity and first step pain (11-point NRS).

### Outcomes and variable selection

The FFI total score at 26 weeks was used as the primary outcome. Secondary outcomes included: the number of patients that reached a minimal important improvement in their total FFI score (an improvement of at least 6.5), the number of patients that considered themselves recovered, the first step pain according to a 11 point NRS and the number of patients that reached a minimal important improvement in their first step pain (an improvement of at least 1.9.) [26, 27] Potential prognostic variables were selected based on supposed clinical relevance and existing literature. The following variables were considered: age, sex, BMI, upper ankle dorsiflexion range of motion in the affected foot, MTP1 dorsiflexion range of motion in the affected foot, navicular drop (ND) in the affected foot, neuropathic pain score in the affected foot (DN4), having bilateral pain, degree of physical activity, reporting other illnesses in the last 12 months, duration of symptoms, FFI scores at baseline and the FPI [22, 25, 28–30]. The FPI was divided into three groups. According to the criteria by Redmond et al. 2006: in patients over 60 years of age, a score between 1 and 8 was considered neutral foot posture, a score below 1 was considered supinated foot posture and a score above 8 was considered pronated foot posture. In patients between 18 and 60 years, a score between 1 and 7 was considered normal foot posture, a score below 1 was considered supinated foot posture and a score above 7 was considered pronated foot posture [25].

### Statistical analysis

If missing values were completely random and the underlying values were normally distributed, they were imputed and if applicable, the minimum and maximum values were assigned as constraints by the researchers. MTP1 joint dorsiflexion (*N* = 12; range, 0-∞), upper ankle joint dorsiflexion (N = 12; range, 0–90), foot posture index (*N* = 8; range, − 12 to12) and FFI total score at 26 weeks (without subscales) (*N* = 16; range, 0–100) were imputed. 50 Imputations and 20 iterations using fully conditional specification (Markov Chain Monte Carlo (MCMC)) were used [31].

Descriptive statistics were used to describe the selected outcome variables and to calculate to number of patients that reached an minimal important difference. All variables were tested for normality of residuals using P-P plots and homoscedasticity was visually checked by inspection of scatterplots of residuals. All variables were checked for a linear relationship with the outcome variable. Multicollinearity was checked using bivariate correlations. First, univariate analyses were performed using linear regression to test the association between each of the potential prognostic factors as described above and the outcomes ((i.e. FFI total score, recovery and first step pain respectively), in patients treated with insoles. The data was checked for outliers, and in case of impossible values, the value was removed. Thereafter, multivariable linear regression analyses with an ENTER model were performed to test the association between the potential prognostic factors as described above and each of the outcomes (i.e. FFI total score, recovery and first step pain respectively) after 26 weeks of follow-up in patients treated with insoles.

Secondly variables with a significant association with the FFI score at 26 weeks in patients treated with insoles were tested for interaction effects using linear regression, with the allocated treatment in patients treated with custom-made insoles versus sham. All analyses were performed using SPSS v25.0.

## Results

Baseline characteristics of the 139 included participants (sham insole group *N* = 69, custom insole group *N* = 70) are presented in Table 1. Table 2 shows the outcomes of participants at 12 and 26 weeks follow-up. Mean total FFI score at baseline was 48.7 for the total population indicating moderate foot function levels. FFI scores improved during follow up (26 weeks) on average with 24 points. At 26 weeks, 79.1% of all participants reached FFI total score MID (an improvement of at least 6.5 points). Women reported higher symptom and lower foot function scores than men at baseline .

**Table 1:** Baseline demographics (*N* = 185)

	Total population *N* = 139 mean (SD) unless otherwise indicated	Sham insole group *N* = 69 mean (SD) unless otherwise indicated	Custom made insole group *N* = 70 mean (SD) unless otherwise indicated
Age, y	48.1 (10.4)	48.2 (9.4)	48.0 (11.3)
Sex, female, N (%)	96 (69.1)	48 (69.6)	48 (68.6)
BMI, kg/m^2^	29.3 (5.3)	29.5 (4.8)	29.2 (5.8)
Pain history			
FFI total score (0–100)	48.2 (18.1)	46.1 (17.2)	50.2 (18.8)
FFI pain score (0–100)	57.8 (17.0)	55.6 (17.2)	60.0 (16.7)
FFI disability score (0–100)	39.5 (21.5)	37.3 (19.7)	41.6 (23.1)
First step pain score (0–10)	7.2 (2.2)	7.3 (2.1)	7.2 (2.4)
Pain at other sites than the affected foot, N (%)	61 (43.9)	32.0 (46.4)	29.0 (41.4)
DN4 score (0–10)	3.8 (2.0)	3.6 (1.8)	3.9 (2.1)
Localization of complaints, bilateral, N (%)	32 (23.0)	16 (23.2)	16 (22.9)
Duration of symptoms, months	6.4 (11.6)	5.1 (5.2)	7.7 (15.5)
Activity			
Squash questionnaire 0 – ∞	7751,1 (5246.0)	8755.3 (5747.8)	6761.3 (4525.5)
Podiatrist measurements			
Upper ankle dorsal flexion range of motion (degrees)	16.42 (1.43)	15.52 (2.24)	17.34 (1.77)
MTP1 dorsal flexion range of motion (degrees)	61.38 (2.09)	61.11 (3.10)	61.65 (2.83)
Pronated foot posture in the affected foot according to the foot posture index, N (%)	39 (28.1)	26 (37.7)	13 (18.6)
Supinated foot posture in the affected foot according to the foot posture index, N (%)	14 (10.1)	7 (10.1)	7 (10.0)

**Table 2:** Outcomes at 12 weeks and 26 weeks follow-up

	Follow-up at 12 weeks			Follow-up at 26 weeks		
	Sham insole group *n* = 68 mean (SD) unless otherwise indicated	Custom made insole group *n* = 66 mean (SD) unless otherwise indicated	Usual care group *N* = 39 mean (SD) unless otherwise indicated	Sham insole group *n* = 68 mean (SD) unless otherwise indicated	Custom made insole group *n* = 66 mean (SD) unless otherwise indicated	Usual care group *N* = 38 mean (SD) unless otherwise indicated
FFI total score, 0–100	30.3 (21.0)	30.8 (23.2)	30.2 (24.6)	24.6 (24.1)	25.0 (23.1)	22.2 (26.5)
FFI total MID*, N (%)	48 (70.6)	45 (68.2)	27 (69.2)	53 (77.9)	53 (80.3)	30 (78.9)
Recovered**, N (%)	25 (36.8)	24 (36.4)	15 (32.6)	39 (57.4)	35 (53.0)	21 (55.3)
First step pain, 0–10	5.0 (3.0)	5.0 (3.0)	4.0 (0.0)	3.0 (3.0)	4.0 (4.0)	3.0 (3.0)
First step pain MID, N (%)***	38 (55.9)	39 (59.1)	23.0 (59.0)	48 (70.6)	43 (65.2)	28 (73.7)

* A patient was considered to have reached a minimal important improvement in FFI if their FFI total score at follow-up was at least 6.5 (the minimal important difference) lower than at baseline

**A patient is defined as recovered if they answered ‘the complaints have completely disappeared or they are strongly improved’

***A patient was considered to have reached a minimal important improvement in first step pain if their first step pain score follow-up was at least 1.9 (the minimal important difference) lower (improved) than at baseline

The univariate analyses in patients treated with insoles **(**Table 3**)** showed that having unilateral pain (β − 13.05 95%CI -22.62,-3.47), lower score on the DN4 for neuropathic pain (β 15.27 95%CI 6.75, 23.78), lower BMI (β 1.25 95%CI 0.53, 1.98), lower baseline FFI score (β .63 95%CI 0.43, 0.84) and lower baseline first step pain score (β 2.04 95%CI 0.30, 3.78) were significantly associated with lower total FFI score at 26 weeks, indicating better outcomes. In the multivariable analysis, only female sex (β − 9.59 95%CI - 17.87;-1.31) and the baseline FFI score (β .56 95%CI 0.30, 0.82) were associated with lower FFI total scores at 26 weeks (R^2^ = 0.543).

**Table 3:** Linear regression analyses of factors associated with FFI total score at 26 weeks follow-up

	Patients treated with insoles (custom + sham)		
	Univariate	Multivariable model	
Variables	Unstandardized β (95% CI)	Unstandardized β (95% CI)	Standardized Beta
Body Mass Index (kg/m^2^)	1.25 (0.53, 1.98)*	0.69 (−0.05, 1.43)	0.16
Comorbidities (present)	−3.43 (− 13.67, 6.81)	−3.86 (− 13.87, 6.15)	− 0.06
Sex (female)	−0.38 (−9.05,8.29)	−9.59 (− 17.87,-1.31)*	0.19
Age (years)	0.08 (− 0.32, 0,47)	0.27 (− 0.13, 0.67)	0.12
Bilateral pain (no)	−13.05 (−22.62, − 3.47)*	−8.41 (− 18.31, 1.50)	0.15
Neuropathic pain DN4 total score (0–10)	15.27 (6.75, 23.78)*	− 0.21 (− 2.34, 1.92)	− 0.02
Upper ankle joint dorsal range of motion (degrees)	− 0.10 (− 0.37, 0.16)	0.02 (− 0.25, 0.28)	0.01
Metatarsal phalangeal joint dorsal range of motion (degrees)	0.06 (− 0.13, 0,24)	0.08 (− 0.08, 0.24)	0.08
Navicular drop (millimeter)	−4.73 (− 14.74, 5.29)	− 3.62 (− 13.12, 5.87)	−0.07
Activity score (SQUASH)	0.0 (0.00, 0.00)	0.00 (0.00, 0.00)	0.10
Treatment group (custom insole)	0.36 (−7.64, 8.35)	0.59 (− 7.11, 8.30)	0.01
Baseline FFI total score (0–100)	0.63 (0.43, 0.84)*	0.56 (0.30, 0.82)*	0.43
Baseline first step pain (0–10)	2.04 (0.30, 3.78)*	−0.01 (− 1.89, 1.70)	− 0.01
Duration of symptoms (months)	0.25 (− 0.09, 0.59)	0.05 (− 0.30, 0.40)	0.02
Foot posture index (supination)	0.05 (−13.45, 13.55)	−1.14 (− 13.68, 11.39)	− 0.02
Foot posture index (pronation)	5.07 (− 3.79, 13.93)	1.32 (−7.35, 10.00)	0.03

*p-value < 0.05

In the patients treated with usual care only variables that gave a significant result in the patients treated with insoles were analyzed

Exploratory analyses in patients treated with insoles showed no significant interaction effects between the allocated treatment (custom-made versus sham) and any of the potential predictive factors (Table 4).

**Table 4:** Interaction between variables of interest and treatment with custom insoles compared to sham insoles for the total FFI score at 26-weeks follow-up

Variables	Unstandardized β (95% CI)	Interpretation
Body Mass Index (kg/m^2^)	0.49 (−1.00, 1.98)	Lower BMI is associated with a lower total FFI score in patients treated with custom insole (non-significant)**
Neuropathic pain DN4 total score (0–10)	1.58 (− 2.56, 5.73)	Lower score on the DN4 score for neuropathic pain is associated with a lower total FFI score in patients treated with custom insole (non-significant)**
Bilateral pain (yes)	−2.16 (− 21.49, 17.16)	Presence of bilateral pain is associated with a lower total FFI score in patients treated with custom insole (non-significant)**
Sex (male)	−12.10 (−29.46, 5.25)	Male sex is associated with a with a lower total FFI score in patients treated with custom insole (non-significant)**
FFI score at baseline	0.02 (−0.39, 0.43)	Lower FFI score at baseline is associated with a lower total FFI score in patients treated with custom insole (non-significant)**
First step pain at baseline	0.47 (−3.07, 4.01)	Lower pain score for first step pain at baseline is associated with a lower total FFI score in patients treated with custom insole (non-significant)**

*Variables were multiplied with a factor indicating treatment (custom insole vs sham)

**A lower total FFI is indicative of a lower pain and better function

## Discussion

All three treatment groups showed improvement over time when compared to baseline, according to the FFI total score, recovery rate and first step pain. Women reported higher symptom and disability scores than men at baseline. Of 16 potential characteristics analyzed in this study, female sex as well as less severe symptoms at baseline were associated to better outcomes at 26 weeks follow-up in patient with PHP being treated with insoles. Of the six characteristics tested for interaction with type of treatment, none had a significant interaction. Although sex had no significant interaction with treatment effect, the confidence interval was wide and included more values on the negative side (95%CI -29.46;5.25). Given the small sample size and the exploratory nature of the present analysis, it is possible this study lacked power to demonstrate interaction effects.

The FFI total score at 26 weeks was chosen as primary outcome because of its clinical relevance and the fact that a high percentage of patients improved by at least 6.5 points (the minimal important difference) at 26 weeks when compared to baseline. The choice for this outcome may have influenced the prognostic variables. In this study the FFI total score improved significantly in both treatment groups. At 26 weeks, 79.1% of all participants reached FFI total score MID, while 55.2% self-reported to be recovered. In literature, recovery rates of 80 to 90% within 10 to 12 months are reported [32]. Follow-up in our study was relatively short at 6 months, which may explain the lower recovery rate when compared to literature. It can be noted that many patients, regardless of a meaningful improved in function, still do not consider themselves recovered.

A high BMI is a known risk factor for the development of PHP and has been found as a prognostic factor for long lasting complaints in patients treated with insoles in a previous study [5, 18, 33]. In the present study there was no significant association between BMI and the total FFI score in the multivariable model. However, BMI was associated with the FFI score in the univariate model. This is likely due to the relatively lower (better) FFI scores at baseline in those with a relatively low BMI and therefore only the FFI score at baseline remained significant in the multivariable model.

PHP is more common among females, however the relationship between sex and prognosis was still unknown [3]. In the present study, both sexes showed general improvement of symptoms, as is expected in a self-limiting disease. Women reported higher symptom and disability scores than men at baseline, but reported similar scores to males at 26 weeks. In the present study sex went from being non-significant in the univariate model to being significant in the multivariable model. The significance of sex changed when the baseline FFI total score was added to the model. Indicating that when the higher score reported by females at baseline (indicating higher disability) is taken into account, sex is a significant prognostic factor.

### Strengths and limitations

To our knowledge, so far only one study has focused on patient characteristics that predict response to treatment with insoles in patients with PHP. A strength of this analysis is the fact that is based on a high-quality randomized trial where we found no differences between custom-made insoles and sham insoles [15]. This means that the context for patients in these two treatment groups was identical and that the data from trial is suitable to assess the prognosis and interaction of insole treatment in patients with PHP.

The main limitation of this analysis is the relatively small sample size of 139 patients. The data in the STAP study was not collected with the primary aim to perform these analyses. This limited the power and the number of potential patient characteristics that could be included. Some confidence intervals were wide and further analysis with higher power may potentially identify significant effects. Moreover, the explained variance of the multivariable model was rather low, with an explained variance of 54%.

There are no studies on prognostic variables for PHP in populations with another treatment than insoles, it therefore remains unknown, whether the prognostic variables found in this study are representative in a different context.

Furthermore, PHP is a condition that can remain symptomatic for over a year in a small percentage of patients [34]. Our follow-up was limited to 26 weeks, not allowing to measure effects on the longer term. Also, 32 participants with bilateral foot complaints were randomized into a ‘left side’ and ‘right side’ group, since data on the MTP range of motion and the navicular drop were only included of the (most) affected foot. The drawback is that for some participants we may have used measurements from the least symptomatic foot, while the FFI scores and self-reported recovery are reported for the most symptomatic foot, increasing chances for a type 2 error.

Finally, PHP is a broad term which can cover a range of different pathologies of heel pain. Given the inclusion criteria of the STAP-study, it is possible that the patients included in this study have a range of different causes of heel pain as we did not include the specific criteria of pain at palpation of the medial tubercle of the calcaneus. Therefore, the exact origin of the participants’ s complaints remains unknown. However, exploring a wide differential diagnosis of PHP is often not indicated, since it does not influence clinical decision making [2]. We therefore believe the population included in this study is representative for the population presenting to the GP with PHP.

## Conclusions

Female sex and less severe initial complaints, in terms of foot function, are associated with lower total FFI score at 26 weeks in patients with PHP who receive treatment with insoles. Future studies should take a difference in response to treatment with insoles for males versus females into account in their design.

## Data Availability

The datasets used and/or analysed during the current study are available from the corresponding author on reasonable request.
